# Association of Tecovirimat Therapy With Mpox Symptom Improvement: A Cross-sectional Study—King County, Washington, May–October 2022

**DOI:** 10.1093/ofid/ofae029

**Published:** 2024-01-22

**Authors:** Ellora N Karmarkar, Matthew R Golden, Roxanne P Kerani, Sargis Pogosjans, Eric J Chow, Rachel A Bender Ignacio, Meena S Ramchandani, Meagan K Kay, Chase A Cannon, Julia C Dombrowski

**Affiliations:** Division of Allergy and Infectious Diseases, Department of Medicine, University of Washington, Seattle, Washington, USA; Division of Allergy and Infectious Diseases, Department of Medicine, University of Washington, Seattle, Washington, USA; Public Health - Seattle & King County HIV/STI/Hepatitis C Program, Seattle, Washington, USA; Division of Allergy and Infectious Diseases, Department of Medicine, University of Washington, Seattle, Washington, USA; Department of Epidemiology, University of Washington, Seattle, Washington, USA; Communicable Disease Epidemiology and Immunization Section, Public Health - Seattle & King County, Seattle, Washington, USA; Division of Allergy and Infectious Diseases, Department of Medicine, University of Washington, Seattle, Washington, USA; Department of Epidemiology, University of Washington, Seattle, Washington, USA; Communicable Disease Epidemiology and Immunization Section, Public Health - Seattle & King County, Seattle, Washington, USA; Division of Allergy and Infectious Diseases, Department of Medicine, University of Washington, Seattle, Washington, USA; Vaccine and Infectious Disease Division, Fred Hutchinson Cancer Research Center, Seattle, Washington, USA; Division of Allergy and Infectious Diseases, Department of Medicine, University of Washington, Seattle, Washington, USA; Public Health - Seattle & King County HIV/STI/Hepatitis C Program, Seattle, Washington, USA; Communicable Disease Epidemiology and Immunization Section, Public Health - Seattle & King County, Seattle, Washington, USA; Division of Allergy and Infectious Diseases, Department of Medicine, University of Washington, Seattle, Washington, USA; Public Health - Seattle & King County HIV/STI/Hepatitis C Program, Seattle, Washington, USA; Division of Allergy and Infectious Diseases, Department of Medicine, University of Washington, Seattle, Washington, USA; Public Health - Seattle & King County HIV/STI/Hepatitis C Program, Seattle, Washington, USA

## Abstract

**Background:**

Data on tecovirimat effectiveness for human mpox are limited. We conducted a retrospective cross-sectional interview-based study to identify associations between tecovirimat treatment and the mpox clinical course.

**Methods:**

Using public health surveillance data from King County, Washington, we recruited and interviewed persons diagnosed with mpox during May–October 2022. We calculated descriptive statistics on demographics, vaccination status, comorbidities, and symptoms including 3 self-reported dates (symptom onset, first date of symptom improvement, and illness resolution). We used multivariable linear regression, stratified by illness severity, to evaluate the association of tecovirimat treatment with time to symptom improvement and time to illness resolution. We compared individuals who did not receive tecovirimat to participants who started tecovirimat early (≤5 days from symptom onset) and late (>5 days and ≤28 days from symptom onset) in their illness.

**Results:**

Of 465 individuals diagnosed with mpox, 115 (25%) participated in this study. Eighty participants (70%) received tecovirimat and 43 (37%) initiated tecovirimat early. Sixty-eight (59%) reported severe symptoms during their illness, including proctitis (n = 38 [33%]), rectal bleeding (n = 27 [24%]), or severe pain (n = 24 [21%]). In the multivariable analysis, early tecovirimat was associated with shorter time to symptom improvement (−5.5 days, *P* = .04) among participants with severe illness but not among those with nonsevere illness (0.9 day, *P* = .66). Early tecovirimat was not associated with faster illness resolution, regardless of severity.

**Conclusions:**

Our small study suggests that early tecovirimat initiation may hasten subjective symptomatic improvement in people with severe mpox. Larger randomized trials are needed to evaluate this finding.

## Teaser

We interviewed 115 persons with mpox who did and did not receive tecovirimat, and performed a multivariable regression analysis. Initiating tecovirimat within 5 days of symptom onset appeared to be associated with faster improvement of symptoms in patients with severe mpox.

During the 2022 mpox outbreak, >30 000 people were diagnosed with mpox in the United States (US) [1]. Tecovirimat, a membrane envelope protein inhibitor that blocks *Monkeypox virus* (MPXV) virion formation and release from host cells, is one of the few available antiviral therapies for mpox [2, 3]. Evidence supporting the potential effectiveness of tecovirimat is derived from studies using animal models, which suggest that tecovirimat slows progression of disease [2–4]. It remains unclear how this potential effectiveness of tecovirimat translates to human illness, as these studies used lethal inoculations of MPXV in animal models and so may not accurately model the drug's impact on human mpox [2–4].

During the 2022 outbreak, constrained access to tecovirimat including challenges with equitable distribution [5, 6] and differences in prescribing practices, and the rapid decrease of MPXV infections in the US and internationally hindered assessment of tecovirimat's effectiveness in real-world settings. Mpox research was poorly funded prior to 2022 despite the burden of disease in endemic areas, and thus data from prospective controlled trials of mpox therapies are limited [6, 7]. Global provision of tecovirimat was limited during the outbreak, even in high-resource settings [8, 9]. Consequently, most available treatment data in humans has been limited to small case series and uncontrolled retrospective observational studies (largest n = 25) [8–15]. Data from these reports suggest that tecovirimat is safe and well-tolerated in patients with mpox, with symptoms improving shortly after treatment is initiated; however, without a control group, tecovirimat effectiveness remains uncertain [8–15]. One controlled observational study of tecovirimat (n = 41) did not detect a difference in time to healing or viral clearance when comparing participants treated with tecovirimat within 10 days of symptom onset to untreated patients [16]. While multiple randomized controlled trials (RCTs) designed to evaluate tecovirimat began during 2022 [17–19], enrollment has been limited.

Within King County, approximately 70% of persons diagnosed with mpox received tecovirimat, with most prescriptions provided by the Public Health–Seattle & King County (PHSKC) Sexual Health Clinic and academic medical centers [20]. The substantial proportion of people receiving tecovirimat presented a unique opportunity to assess associations between tecovirimat treatment and duration of mpox symptoms. Timing of treatment was of interest, as prior literature suggests that early treatment for other viral illnesses (ie, severe acute respiratory syndrome coronavirus 2 [SARS-CoV-2], Ebola) is more effective than treatment initiated later in the course [21–23]. Given the urgent need to determine potential benefits of tecovirimat during this worldwide outbreak, we conducted a retrospective cross-sectional interview-based study to assess for associations between tecovirimat treatment and self-reported mpox symptom improvement and illness resolution among King County residents diagnosed with mpox between May and October 2022. We evaluated the association of the timing of tecovirimat initiation (within 5 days of symptom onset and >5 days from symptom onset) with time to symptom improvement and time to illness resolution.

## METHODS

### Patient Consent Statement

Verbal informed consent was obtained by phone. Participants were informed of the purpose of the study, how data would be stored, that the study was voluntary and they could stop the interview at any time, and that not participating would not affect their care at any healthcare institution. Participants verbally consented to participation before the study-related interview began. The study was approved by the University of Washington Institutional Review Board (STUDY00016261), and requirement for written consent was waived.

### Study Population

We used PHSKC mpox surveillance data to identify potential participants. Mpox is an immediately notifiable condition in King County, and PHSKC receives reports of positive mpox real-time polymerase chain reaction (rtPCR) testing from the Washington State Public Health Laboratory, local academic and private laboratory services, and clinical providers. Trained PHSKC disease investigators attempt to contact or abstract medical records on all persons with suspected or laboratory-confirmed mpox to gather demographic data (including race and ethnicity, gender, age) and contact information for entry into the PHSKC mpox database.

Individuals were eligible for the study if they were King County residents with laboratory-confirmed mpox between May and October 2022, had complete resolution of illness at time of interview, and were in the PHSKC mpox surveillance database.

#### Recruitment

Recruitment was conducted using the contact information available in the PHSKC database. We attempted to contact all potential participants by phone, email, and text, with at least 3 attempts made for each person. The primary investigator and a research assistant conducted recruitment and all interviews between November 2022 and February 2023. Persons who consented to participation in the study were interviewed by phone using a semi-structured questionnaire. For Spanish-speaking participants, a medical translator was used. Participants were compensated US$15 for participation.

### Data Collection

Study data were collected and managed using REDCap (Research Electronic Data Capture) [24, 25]. We used semi-structured interviews to collect patient self-reported information on demographics (age; participant-identified gender and sexual orientation, including cisgender men who have sex with men [MSM], transgender persons, and persons who identify as nonbinary/genderqueer; race/ethnicity; gender of sexual partners; level of education), mpox vaccination status, comorbidities (human immunodeficiency virus [HIV] status, eczema or psoriasis, heart disease), mpox clinical symptoms, and illness severity. We used structured data fields and collected free-text descriptions if participant responses did not conform to the available response options.

Interviewers collected information on the clinical course using participant recall or estimation of key dates (onset date, date of symptom improvement, date of illness resolution). Given risk of recall bias, the interview was structured to ask about symptoms and illness course prior to asking if the participant was prescribed tecovirimat. Participants were provided their specific mpox testing date recorded in the PHSKC database to help prompt their memory of other key dates. They were advised to refer to a calendar, photos, or their personal medical records or notes that could help them remember key dates in their illness course during the interview. We did not consent patients for formal medical record review to maintain a low barrier for participation in our study. We clarified whether participants had started tecovirimat, and if so, the date they started and whether they received tecovirimat prior to test results (presumptive) or after results returned (results-based). We also asked about side effects to tecovirimat.

#### Key Variable Definitions

Symptom onset date was defined as the date that the participant first noticed any symptom of illness attributable to mpox (specifically fevers, chills, malaise, or lesions).

Symptom improvement date was elicited by asking the date that the participant had any subjective improvement in these symptoms, which could include “beginning to feel better,” resolution of fever or chills, decreased pain of lesions, or any other indication of improving health.

Illness resolution date was defined as the date that all mpox symptoms resolved, with completely healed lesions and a new layer of skin.

Time to symptom improvement and illness resolution was calculated as the number of 24-hour periods between the participant-reported onset date and date of symptom improvement or illness resolution.

Severe illness was defined as severe pain (requiring narcotics or lidocaine); rectal bleeding; proctitis or tenesmus; phimosis; difficulty swallowing; ≥100 lesions; hospitalization; or patient-reported clinician concern for bacterial superinfection. This reflected the definitions used by the Centers for Disease Control and Prevention (CDC) [26] and the Study of Tecovirimat for Human Monkeypox Virus (STOMP) trial (NCT05534984) [17].

### Key Predictors and Outcomes

The primary predictor of interest was whether the participant started tecovirimat. The secondary analysis was based on time from symptom onset to tecovirimat initiation. We dichotomized time to tecovirimat into early tecovirimat (within 5 days of symptom onset), or late tecovirimat (>5 days and up to 28 days from symptom onset). The category of early tecovirimat was selected based on definitions of early treatment for other viral infections and for symmetry with the planned analyses in the STOMP trial (NCT05534984) [17]. We chose an upper limit of 28 days for the tecovirimat late initiation category, given the average maximum duration for mpox illness of 4 weeks reported by the CDC [27]. Receipt of tecovirimat and timing of initiation were based on participant report. We analyzed on intention to treat, defined as a participant reporting tecovirimat start, whether or not the course was completed. Our primary outcome of interest was time to complete illness resolution from onset date; the secondary outcome of interest was time to symptom improvement from onset date.

### Analysis

We conducted a descriptive analysis of demographic and clinical variables. We assessed median time (with interquartile range [IQR]) to illness resolution and symptom improvement based on ever receiving tecovirimat, starting tecovirimat early, or starting tecovirimat later. We assessed differences in median time to illness resolution and symptom improvement using Kruskal-Wallis tests.

We stratified analyses by severity of disease given severity might mediate time to improvement and resolution, and we sought to capture any meaningful clinical benefit of treatment for patients with severe viral illness, similar to studies on SARS-CoV-2 and influenza [21, 28]. We created a multivariable linear regression model evaluating association between time to tecovirimat treatment (compared to no tecovirimat) and time to illness resolution or time to symptom improvement. We selected additional covariates a priori with a plausible relationship between both tecovirimat receipt and the timing of participant-reported subjective improvement or resolution. These included self-reported comorbidities such as HIV, eczema or psoriasis, heart disease, diabetes mellitus, or cancer; age as a continuous variable; race and ethnicity; gender and sexual orientation; and level of education (no high school, high school or trade school, college, and graduate education). We included vaccination status given vaccine receipt may modulate susceptibility to illness, severity of illness, and response to tecovirimat. We categorized vaccination receipt into postexposure (at most 7 days prior to symptom onset), partial (1 vaccination >7 days prior to symptom onset), or full vaccination [29] (2 doses of vaccination 2 weeks prior to onset of illness). To prevent model overfitting, dichotomous variables with <10 participants in a single stratified category were excluded from the model, except for gender and race and ethnicity, where we compared the predicted *R*^2^ to the actual *R*^2^ to determine fit of the model. Analyses were conducted using R Studio version 2022.07.2 (R Foundation for Statistical Computing, 2018) Statistical Software; significance was set at *P* < .05 [30].

Our preanalysis power calculations suggested that at a 95% confidence level and 80% power, assuming a time to symptom improvement of at least 10–14 days based on limited available data, we would need 27 patients in each group to detect a difference of 4 days in time to symptom improvement or illness resolution.

## RESULTS

### Demographic and Clinical Data

A total of 465 individuals with laboratory-confirmed mpox during 22 May**–**24 October 2022 met inclusion criteria. Based on PHSKC mpox surveillance data, 443 (95%) were cisgender men; 205 (44%) persons identified as non-Hispanic White, 118 (25%) as Hispanic/Latinx, 45 (10%) as non-Hispanic Black, 26 (6%) as Asian, and 16 (3%) as American Indian/Alaska Native, and 55 (12%) answered “other” or did not disclose race or ethnicity. Of 465 individuals, 311 (67%) were reported to have received tecovirimat, and there were no differences in tecovirimat receipt by race or ethnicity [20].

Among the 465 individuals contacted for interview, 171 (37%) were reached and 115 (25% of total [67% of those successfully reached]) consented to study participation. Sixteen (3%) individuals verbally declined, 40 (9%) did not answer phone calls at the time of scheduled interview, and 294 (63%) were never successfully contacted. Of 350 nonparticipants, based on surveillance data, 146 (42%) were non-Hispanic White, 88 (25%) were Hispanic/Latinx, 34 (10%) were non-Hispanic Black, 48 (14%) answered other or did not disclose race or ethnicity, and 35 (10%) were Asian or American Indian/Alaska Native. Of the 115 participants who all confirmed their race and ethnicity, 58 (50%) were non-Hispanic White, 26 (23%) were Hispanic/Latinx, and 17 (15%) identified as multiracial (Table 1).

**Table 1.** Demographics and Comorbidities of Interview Participants Diagnosed With Mpox (May–October 2022), Seattle–King County, Washington^a^

Demographics and Comorbidities	N = 115
Age, y, median (IQR)	36 (29–42)
Race/ethnicity, No. (%)	
Non-Hispanic White	58 (50.4)
Hispanic/Latinx	26 (22.6)
Multiple races	17 (14.8)
Black or African American	n ≤ 10 (7.8)
Asian or South Asian	n ≤ 10 (4.3)
Education. No. (%)	
Less than high school	n ≤ 10 (0.9)
High school diploma	14 (12.2)
Trade school certificate	n ≤ 10 (7.0)
Community college or university degree	63 (54.8)
Graduate degree	25 (21.7)
Other	n ≤ 10 (3.5)
Gender, No. (%)	
Cisgender male	103 (89.6)
Gender diverse^b^	12 (10.4)
Sexual partners (not mutually exclusive), No. (%)	
Cisgender male	110 (95.7)
Transgender male	12 (10.4)
Nonbinary/genderqueer	10 (8.7)
Cisgender female	n ≤ 10 (5.2)
Transgender female	n ≤ 10 (4.3)
Other	n ≤ 10 (3.5)
Comorbidities, No. (%)	
None of the below comorbidities	53 (46.9)
HIV	38 (33.6)
CD4 count ≥200 cells/μL	18 (47.3)
CD4 count <200 cells/μL	2 (5.2)
CD4 count unknown	18 (47.3)
Eczema/psoriasis	13 (11.5)
Heart disease	n ≤ 10 (3.5)
Diabetes	n ≤ 10 (1.8)
Cancer	n ≤ 10 (1.8)

Abbreviations: HIV, human immunodeficiency virus; IQR, interquartile range.

^a^Small cell sizes ≤10 are suppressed.

^b^Gender diverse includes nonbinary/genderqueer, transgender men and women, and other.

Most participants were cisgender men (90% [n = 103]) and had male sexual partners (96% [n = 110]). Thirty-eight (33%) persons were living with HIV; 18 (47%) had CD4 counts ≥200 cells/μL, 2 (5%) had CD4 counts <200 cells/μL, and 18 (47%) did not recall their CD4 count. Eighty-eight (77%) had a college or graduate degree.

Nearly 60% of study participants (n = 68 [59%]) had severe mpox, including rectal pain/tenesmus/proctitis (n = 38 [33%]), rectal bleeding (n = 27 [23%]), and severe pain (n = 24 [21%]), not mutually exclusive (Table 2). Approximately one-third of participants (n = 37 [32%]) received at least 1 vaccine dose before symptom onset. Eighteen (16%) received vaccine up to a week prior to symptom onset, 17 (15%) received at least 1 vaccine >1 week prior to symptom onset (partially vaccinated), and only 2 (2%) were fully vaccinated prior to symptom onset. Of 78 participants who did not receive vaccine prior to symptom onset, 49 (63%) developed severe disease, compared to 11 of 18 (61%) participants who received vaccine within 7 days prior to symptom onset, 7 of 17 (41%) participants who were partially vaccinated, and 1 of 2 (50%) fully vaccinated participants. Most participants (n = 80 [70%]) received tecovirimat, with 52 of 80 (65%) receiving tecovirimat after positive rtPCR testing and 28 (35%) receiving presumptive treatment. Twenty-five (31%) reported side effects from tecovirimat, with headache as the most common side effect (n = 10 [13%]). The median time to tecovirimat initiation was 5 days from symptom onset (IQR, 3–8.5 days), and 43 of 80 (54%) participants initiated tecovirimat within 5 days of symptom onset.

**Table 2.** Self-reported Clinical Characteristics of Interview Participants Diagnosed With Mpox (May–October 2022), Seattle–King County, Washington

Mpox Clinical Characteristics	No. (%)
Symptoms^a^	
Anal or genital sores	84 (73.0)
Fatigue	77 (67.0)
Fevers/chills	66 (57.4)
Sores or bumps on trunk, extremities	58 (50.4)
Sores in multiple places	33 (28.9)
Sores or bumps on the face	31 (27.0)
Sore throat	30 (26.1)
Severe symptoms^a^	
Rectal pain/tenesmus/proctitis	38 (33.0)
Rectal bleeding	27 (23.5)
Severe pain requiring lidocaine or narcotics	24 (20.9)
Penile swelling or phimosis	21 (18.3)
Difficulty swallowing	14 (12.2)
Concern for superinfection	11 (9.6)
>100 lesions	n ≤ 10 (2.6)
Hospitalization	n ≤ 10 (2.6)
Scarring	47 (40.9)
Mpox vaccination	
Unvaccinated	55 (47.8)
Received at least 1 vaccination	60 (52.2)
Vaccinated after onset of illness	23 (20.0)
Received 1 vaccine within 7 d prior to onset (PEP)	18 (15.6)
Received 1 vaccine ≥1 wk before onset	17 (14.8)
Fully vaccinated before onset	2 (1.7)
Tecovirimat therapy	
Received tecovirimat	80 (69.6)
Received tecovirimat ≤5 d from onset (n = 80)	43 (53.8)
Results-based therapy (n = 80)	52 (65.0)
Presumptive therapy (n = 80)	28 (35.0)
Completed 14 d of therapy (n = 80)	74 (92.5)
Side effects of therapy^a^	
Any side effect	25 (31.3)
Headache	10 (12.5)
Nausea	9 (11.3)
Abdominal pain	6 (7.5)
“Mental fog”	6 (7.5)
Dizziness	6 (7.5)
Diarrhea	5 (6.3)

Abbreviation: PEP, postexposure prophylaxis.

^a^Not mutually exclusive.

### Median Time to Illness Resolution and Symptom Improvement

In analyses stratified by illness severity, among participants with severe disease, median time to illness resolution among those receiving tecovirimat was 20 (IQR, 14–28) days compared to 21 days (IQR, 18–31) among those not receiving tecovirimat (*P* = .56; Table 3). There was no significant difference in time to illness resolution for participants receiving tecovirimat early (median, 18 [IQR, 12–26] days; *P* = .11) or late (median, 26 [IQR, 17–28] days; *P* = .59) compared to untreated participants. Median time to symptom improvement was 11 (IQR, 7–16) days with tecovirimat and 15 (IQR, 10–18) days without tecovirimat receipt, respectively (*P* = .28). Median time to symptom improvement did differ with early tecovirimat (8 [IQR, 6–13] days; *P* < .01) compared to no tecovirimat, but not with later tecovirimat compared to no tecovirimat (16 [IQR, 10–23] days; *P* = .49).

**Table 3.** Difference in Median Days to Symptom Improvement and to Illness Resolution by Tecovirimat Receipt by Kruskal-Wallis Testing, Stratified by Severity of Disease Among Participants Diagnosed With Mpox (May–October 2022), Seattle–King County, Washington

Tecovirimat Status	Median Days to Improvement (IQR)	Difference in Days (*P* Value)	Median Days to Resolution (IQR)	Difference in Days (*P* Value)
Severe disease (n = 68)				
No tecovirimat (n = 20)	15 (10–17.8)	Referent	21 (17.5–30.5)	Referent
Any tecovirimat (n = 48)	10.5 (7.0–16.0)	−4.5 (.28)	20 (14.0–27.5)	−1 (.56)
Tecovirimat within 5 d of symptom onset (n = 24)	7.5 (5.8–13.2)	−7.5 (<.01)^a^	17.5 (12.0–26.2)	−3.5 (.11)
Tecovirimat >5 d and <28 d after symptom onset (n = 19)	16 (10.0–22.5)	1 (.49)	26 (16.5–28.0)	5 (.59)
Nonsevere disease (n = 47)				
No tecovirimat (n = 15)	8.5 (5.25–11.8)	Referent	17 (13.2–26.2)	Referent
Any tecovirimat (n = 32)	9 (7.0–15.0)	.5 (.32)	23 (16.0–28.0)	6 (.19)
Tecovirimat within 5 d of symptom onset (n = 19)	9 (7.0–13.0)	.5 (.57)	22 (14.0–27.5)	5 (.56)
Tecovirimat >5 d and <28 d after symptom onset (n = 11)	11 (7.5–15.5)	2.5 (.35)	23 (21.0–27.0)	6 (.07)^b^

Abbreviation: IQR, interquartile range.

^a^Kruskal-Wallis *P* < .05.

^b^Kruskal-Wallis *P* < .10.

For participants without severe disease, there was no significant association between median time to symptom improvement based on tecovirimat receipt (*P* = .32) or time to tecovirimat receipt (*P* = .57 for early tecovirimat, *P* = .35 for later tecovirimat). There was also no significant association between median time to illness resolution based on tecovirimat receipt (*P* = .19) or time to tecovirimat receipt (*P* = .56 for early tecovirimat, *P* = .07 for later tecovirimat) (Table 3).

### Multivariable Linear Regression

In the multivariable analysis, among participants with severe disease, receiving tecovirimat was not significantly associated with time to illness resolution (Table 4). However, receiving tecovirimat early was associated with faster time to symptom improvement (−5.5 days; *P* = .04).

**Table 4.** Multivariable Linear Regression Results Stratified by Disease Severity for Receipt of Early Tecovirimat (≤5 Days From Symptom Onset) and Receipt of Later Tecovirimat (>5 and <28 Days From Symptom Onset) Compared to No Tecovirimat as a Predictor of Time to Illness Resolution and Time to Symptom Improvement in Days

Variable	Time to Illness Resolution		Time to Symptom Improvement	
	Severe Disease	Nonsevere Disease	Severe Disease	Nonsevere Disease
Constant	20.6 (.22)	3.5 (.67)	11.7 (.17)	17.5 (<.01)
No tecovirimat (referent)				
Early tecovirimat^a^	−8.7 (.11)	−0.6 (.85)	−5.5 (.04)^c^	0.9 (.66)
Later tecovirimat^b^	−0.1 (.98)	6.7 (.07)^d^	1.7 (.55)	3.9 (.11)
Age	0.4 (.22)	0.5 (<.01)^c^	0.1 (.44)	−0.05 (.68)
MSM vs gender diverse	−0.7 (.93)	−7.7 (.08)^d^	−2.6 (.5)	−7.8 (<.01)^c^
Race/ethnicity				
Non-Hispanic White (referent)				
Latinx	−1.7 (.78)	0.7 (.83)	−1.0 (.73)	−2.7 (.25)
Black/African American	11.7 (.19)	−2.0 (.72)	2.6 (.54)	−7.8 (0.04)^c^
Multiracial or Asian	−2.7 (.67)	0.4 (.94)	−0.6 (.85)	−4.2 (.18)
PEP or vaccination	−0.5 (.87)	−2.4 (.15)	−0.2 (.92)	−2.2 (0.06)^d^
HIV	−0.4 (.94)	−3.6 (.35)	−1.0 (.69)	0.1 (.96)
Level of education	−2.7 (.57)	4.4 (.12)	1.1 (.64)	1.8 (.35)

Data are presented as coefficient estimate in days (*P* value).

Abbreviations: HIV, human immunodeficiency virus; MSM, men who have sex with men; PEP, postexposure prophylaxis.

^a^Early tecovirimat: initiated within 5 days of symptom onset.

^b^Later tecovirimat: initiated >5 days but <28 days from symptom onset.

^c^Covariate *P* < .05.

^d^Covariate *P* < .10.

For participants without severe disease, increasing age was associated with slower time to illness resolution (0.5 days with each year of age; *P* < .01), and identifying as MSM or Black was associated with shorter time to symptom improvement (−7.8 days, *P* < .01 and −7.8 days, *P* = 0.04, respectively). Other races and ethnicities and educational status were not statistically significant variables in any of the models.

## DISCUSSION

In this county-wide cross-sectional retrospective interview study, we found that initiation of tecovirimat within 5 days of mpox symptom onset among participants with severe disease was associated with faster subjective symptom improvement (−5.5 days; *P* = .04) but did not hasten complete illness resolution. Tecovirimat treatment was not associated with faster symptom improvement or illness resolution in people with nonsevere disease.

Prior studies with smaller patient populations have anecdotally reported symptom improvement with tecovirimat, but they lacked a control group for comparison [8–15]. Our findings reflect outcomes in a relatively large study population of 115 patients, evaluated at the height of the US mpox epidemic in a jurisdiction with a high level (~70%) of treatment. In addition, the multivariable analysis was performed within a stratified population of 68 patients with severe disease including a comparison population without treatment.

Our finding that tecovirimat treatment overall was not statistically associated with time to complete illness resolution is consistent with a recent study that concluded that tecovirimat receipt within 10 days of symptoms onset had no association with improved viral clearance or illness resolution [16]. However, the recent study's inclusion of participants with a long duration of illness prior to treatment may obscure evidence of association between early initiation and illness resolution. Early initiation of tecovirimat in the setting of severe disease may in fact lead to faster illness resolution, though the effect did not reach statistical significance in our study (−8.7 days; *P* = .11) due to small numbers in stratified groups and limited power, or potentially from tecovirimat slowing progression but not hastening resolution. RCTs are needed to clarify this.

Given the potential benefit of early treatment in severe disease, providers could consider offering tecovirimat prior to laboratory confirmation, particularly as data suggest that well-trained sexual health clinic providers can accurately diagnose mpox prior to laboratory confirmation [31]. However, treatment decisions should be integrated with the need for more rigorous data on tecovirimat treatment effectiveness, and patients should be given the opportunity to participate in RCTs as part of the treatment discussion [6].

With regards to vaccination, only 2 participants were fully vaccinated, and only 1 participant who was fully vaccinated and 7 participants who were partially vaccinated developed severe disease, consistent with prior literature describing the effectiveness of vaccine in preventing mpox illness [32–34]. While the small numbers prevent robust analysis of the potential interactions between vaccination and tecovirimat, none were identified in the multivariable model.

Global access to tecovirimat remains challenging [6, 7], and there are persistent disparities in mpox acquisition and access to care among racial and ethnic minority and gender minority groups [1, 35]. Prior analyses on tecovirimat prescriptions in King County did not detect any differences in tecovirimat receipt by race or ethnicity [20]. Our analysis did identify differences in time to symptom improvement in non-severe disease among gender minorities (transgender or nonbinary/genderqueer persons) compared to cisgender MSM, and among persons identifying as Black. These results likely reflect model overfitting from small sample sizes or true variations in mpox-focused care by race, ethnicity, and gender. While the generalizability of our findings is unclear, public health responses should continue to optimize access to care and address disparities among high-risk groups [6, 7, 36].

Our results suggest that for nonsevere disease, older age may be associated with slower time to recovery. Other viral illnesses can have atypical presentations or prolonged illness in older populations [37]. Older adults with mpox were also more likely to be hospitalized [38], which may also suggest prolonged or slow recovery.

Our study has both strengths and limitations. We studied a substantially larger number of patients than prior studies of tecovirimat, enrolled a population-based sample, and included systematic data collection. However, our study may be affected by recall bias, particularly given the time elapsed between mpox illness and interview. Despite our efforts to mitigate this, participants may have trouble recalling key dates, resulting in inaccurate calculations of time to symptom improvement and illness resolution.

We did not collect laboratory correlates of mpox illness and could not compare our interview data directly to medical record data, leading to missingness in HIV data, inability to model HIV status, and limiting the interpretation of our study's findings. Worldwide, many patients with mpox presented with concomitant sexually transmitted infections (STIs) and likely received STI-specific therapy that improved their symptoms [39]; unfortunately, we did not systematically collect STI testing data, which may bias the results of our study. Tecovirimat prescriptions in King County were heterogeneous and we could not determine the level of access to tecovirimat at each site; however, prescribing site and related services may be correlated with availability of tecovirimat [20]. Only 25% of eligible persons completed the interviews, which primarily reflected our team's inability to reach them to offer the study. Without information on severity of illness or clinical course among nonrespondents, we cannot predict how this may have biased our findings. It is reassuring that our study population had a similar proportion with access to tecovirimat (∼70%) as the county-wide population.

Our study population was enriched with college-educated participants and participants who were non-Hispanic White compared to the overall King County population with mpox. Thus, our participants reflect a population that may have better access to care and ability to advocate for themselves, preventing identification of racial and socioeconomic disparities within our study and limiting generalizability. While these limitations are important to consider, our study provides results that may not be obtainable by RCT in the setting of limited cases and a highly effective vaccine [32–34].

In summary, our study suggests that early receipt of tecovirimat within 5 days of symptom onset may be associated with faster subjective symptomatic improvement in patients with severe disease. We did not detect a statistically significant association between treatment and time to complete resolution of illness in patients with severe disease, or either outcome in patients without severe disease. However, RCTs are urgently needed to further explore these findings given the retrospective nature of our study. The ongoing STOMP trial and other international trials will provide critical evaluation of the association of tecovirimat and mpox illness course to inform clinical guidelines for the use of tecovirimat [17–19, 40, 41]. Clinicians should seek to enroll patients with mpox in these trials. When that is not possible, our data suggest that early initiation of tecovirimat in patients with severe disease may be beneficial.

## Notes

***Financial support.*** E. N. K. is supported under National Institutes of Health STD and AIDS Training grant 5T32AI007140–45. Incentives were provided by the King Holmes Endowed Professorship held by J. C. D. The REDCap instance used is supported by the Institute of Translational Health Sciences, which is funded by the National Center for Advancing Translational Sciences of the National Institutes of Health (award number UL1TR002319). All other authors participated in this work as part of their normal duties and did not receive additional funding for this work.
